# Variability of the coupling of blood flow and oxygen metabolism responses in the brain: a problem for interpreting BOLD studies but potentially a new window on the underlying neural activity

**DOI:** 10.3389/fnins.2014.00139

**Published:** 2014-06-11

**Authors:** Richard B. Buxton, Valerie E. M. Griffeth, Aaron B. Simon, Farshad Moradi

**Affiliations:** Department of Radiology, Center for Functional MRI, University of CaliforniaSan Diego, La Jolla, CA, USA

**Keywords:** cerebral blood flow (CBF), cerebral metabolic rate of oxygen (CMRO_2_), blood oxygenation level dependent (BOLD), functional magnetic resonance imaging (fMRI), inhibitory/excitatory neural activity

## Abstract

Recent studies from our group and others using quantitative fMRI methods have found that variations of the coupling ratio of blood flow (CBF) and oxygen metabolism (CMRO_2_) responses to a stimulus have a strong effect on the BOLD response. Across a number of studies an empirical pattern is emerging in the way CBF and CMRO_2_ changes are coupled to neural activation: if the stimulus is modulated to create a stronger response (e.g., increasing stimulus contrast), CBF is modulated more than CMRO_2_; on the other hand, if the brain state is altered such that the response to the same stimulus is increased (e.g., modulating attention, adaptation, or excitability), CMRO_2_ is modulated more than CBF. Because CBF and CMRO_2_ changes conflict in producing BOLD signal changes, this finding has an important implication for conventional BOLD-fMRI studies: the BOLD response exaggerates the effects of stimulus variation but is only weakly sensitive to modulations of the brain state that alter the response to a standard stimulus. A speculative hypothesis is that variability of the coupling ratio of the CBF and CMRO_2_ responses reflects different proportions of inhibitory and excitatory evoked activity, potentially providing a new window on neural activity in the human brain.

## The challenge of interpreting the BOLD response in a quantitative way

Functional magnetic resonance imaging (fMRI) based on the detection of blood oxygenation level dependent (BOLD) signal changes has had an enormous influence on human neuroscience studies, providing a sensitive and noninvasive tool for detecting a change in neural activity in response to a stimulus or during spontaneous neural fluctuations. The basic physical phenomenon underlying the BOLD effect is that deoxyhemoglobin is paramagnetic, and its presence reduces the MR signal slightly (Buxton, 2013). If the blood becomes more oxygenated, the MR signal goes up. Note, though, that this phenomenon by itself is not enough to explain why the BOLD effect happens: one could easily imagine that CBF and CMRO_2_ increase by the same fraction in response to neural activity changes, which would not change blood oxygenation. The existence of the BOLD effect depends also on a second, physiological phenomenon: when neural activity increases CBF increases much more than CMRO_2_—*decreasing* the local oxygen extraction fraction—and the decreased concentration of deoxyhemoglobin creates the BOLD response. While it is widely understood that the BOLD response is not directly related to neural activity, there is nevertheless a tendency to think of it as a relatively simple two-step process: increased neural activity leads to a CBF change, which then produces a BOLD signal change. In this perspective article we argue that this view is too simplistic, because it leaves out the important role played by CMRO_2_: when neural activity increases, the CBF increase tends to wash out deoxyhemoglobin, while the CMRO_2_ increase tends to create more deoxyhemoglobin. For this reason, the BOLD signal depends strongly on the coupling ratio *n*, the ratio of the fractional changes in CBF and CMRO_2_. For example, the same change in CBF will produce a larger BOLD response when *n* is large.

For this reason, interpreting the BOLD response in terms of the underlying neural activity is not just a question of understanding neurovascular coupling; we must also understand neuro-metabolic coupling. Local neural activity includes both synaptic and spiking activity, and both excitatory and inhibitory activity. The basic problem is that we currently do not have a good quantitative understanding of how each of these aspects of neural activity drives CBF and CMRO_2_. Current thinking is that the acute CBF response to a stimulus is not driven directly by the change in energy metabolism, but rather by signals related to the neural activity itself (Attwell and Iadecola, 2002). This essentially feed-forward mechanism provides a way to avoid a potentially dangerous drop in tissue O_2_ concentration by increasing CBF in anticipation of a greater need for oxygen (Buxton, 2010). The need for a relatively fast CBF response is that there is very little O_2_ available in tissue to serve as a buffer [tissue O_2_ in gray matter would be depleted in about 1 s for normal CMRO_2_ (Buxton, 2010)], and a quick increase in CMRO_2_ could lead to a sharp drop in available O_2_ in the tissue unless CBF also quickly rises. This means that we must think of CBF and CMRO_2_ as being driven in parallel by neural activity, but potentially by different aspects of that activity.

These physiological considerations emphasize the difficulty of interpreting the BOLD response in a quantitative way. Most fMRI investigators would support the view that if a local BOLD signal change is detected in response to a stimulus, it suggests that there is some underlying change in neural activity, the basis of using the BOLD response as a mapping signal. However, if we focus on questions comparing BOLD responses under different conditions, the interpretation becomes more problematic: does a change of the underlying neural activity in response to a stimulus necessarily lead to a BOLD signal change? Or, if the BOLD response is different comparing two conditions, does the magnitude of the difference reflect the magnitude of the underlying physiological differences? These are more difficult questions to answer, and reflect a key shift from simply asking where activation occurs to asking how much activation occurs. The difficulty in making this shift is part of the reason for the lack of clinical impact of fMRI, despite the clear potential to provide information on brain dysfunction. The most established fMRI application in a clinical setting is in pre-surgical planning (Chakraborty and McEvoy, 2008), where the basic question is with regard to the location of activity, reflecting the success of fMRI as a mapping tool. For many clinical and neuroscience applications, though, the part of the brain of interest is already known, and the important question is: what is the level of neural activity of that brain area under different conditions?

We take this as the fundamental challenge for fMRI: how can we interpret the magnitude of the BOLD signal in a quantitative way in terms of the underlying physiological activity? Based on the studies discussed below, our conclusion is that the BOLD response alone is ambiguous, and cannot be interpreted reliably as a quantitative reflection of the underlying physiology. Fortunately, though, the combination of BOLD imaging with arterial spin labeling (ASL) methods and a calibrated BOLD approach makes it possible to isolate the effects of CBF and CMRO_2_ (Davis et al., 1998; Hoge, 2012; Pike, 2012). This quantitative fMRI approach provides a much richer context for assessing the underlying physiology of brain activation and offers the potential of revealing more about the underlying neural activity than BOLD imaging alone.

## The complexity of the BOLD response

**Figure 1:**
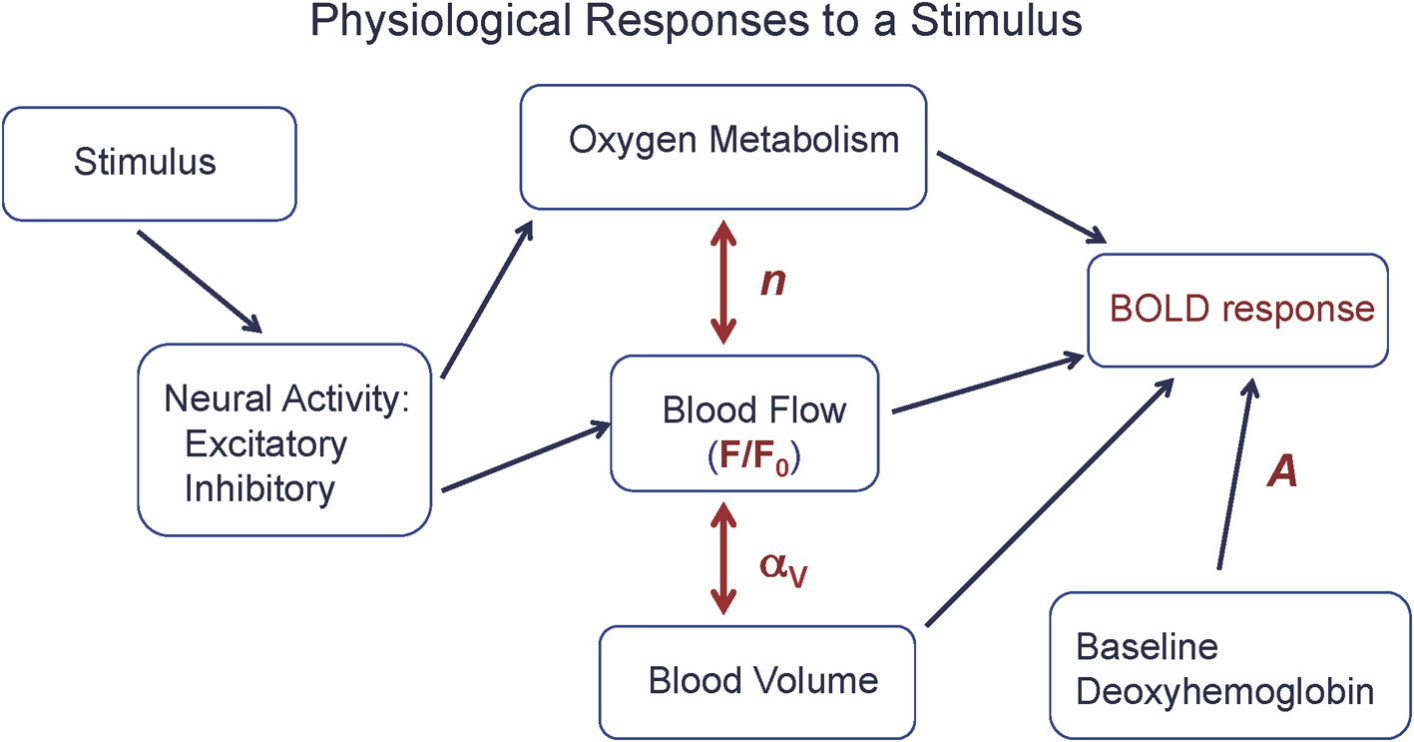
**The physiological basis of the BOLD response**. A stimulus evokes increased excitatory and inhibitory neural activity, with the energy cost of the net evoked activity met primarily by an increase in oxygen metabolism (CMRO_2_), with increased blood flow (CBF) driven by aspects of the neural response. The BOLD response is primarily driven by the CBF change (*F*/*F*_0_), but strongly modulated by the ratio *n* of the fractional changes in CBF and CMRO_2_ and the baseline state (*A*), and to a lesser degree by venous blood volume changes (α_*V*_). Equation (1) is a simple model for the BOLD response in terms of these physiological changes.

From a quantitative viewpoint, we can look at the BOLD response as driven by a CBF change, but strongly modulated by two additional physiological factors: the CBF/CMRO_2_ coupling ratio *n*, discussed above, and the amount of deoxyhemoglobin present in the baseline state (Figure 1). In order to clarify the complexity of the BOLD signal, we introduced a simple heuristic model for the BOLD response (Δ*S*), based on a more detailed model (Griffeth and Buxton, 2011), that approximately captures the different factors involved (Griffeth et al., 2013):

(1) $$\Delta S=A\left(1-1/n-\alpha_{V}\right)\left(1-F_{0}/F\right)$$

The scaling factor *A* is proportional to the total amount of deoxyhemoglobin present in the baseline state, and so depends on the baseline oxygen extraction fraction and venous blood volume, and also depends on technical factors related to the data acquisition (magnetic field strength and echo time). The baseline CBF is denoted *F*_0_, and the activated CBF is denoted *F*. The nonlinear dependence on *F* reflects the ceiling effect on the BOLD response: even a very large flow is limited in its effect because it can only reduce the finite amount of deoxyhemoglobin present in the baseline state. The parameter α_*V*_ describes the effect of a change in venous blood volume with activation, which changes the total blood volume containing deoxyhemoglobin. Typical values of the parameters for a strong activation in visual cortex are *A* = 0.12, *F*/*F*_0_ = 1.4 (40% flow increase), *n* = 2 (20% CMRO_2_ increase), and α_*V*_ = 0.2 (Chen and Pike, 2009), giving a BOLD signal change of about 0.01 (1%).

**Figure 2:**
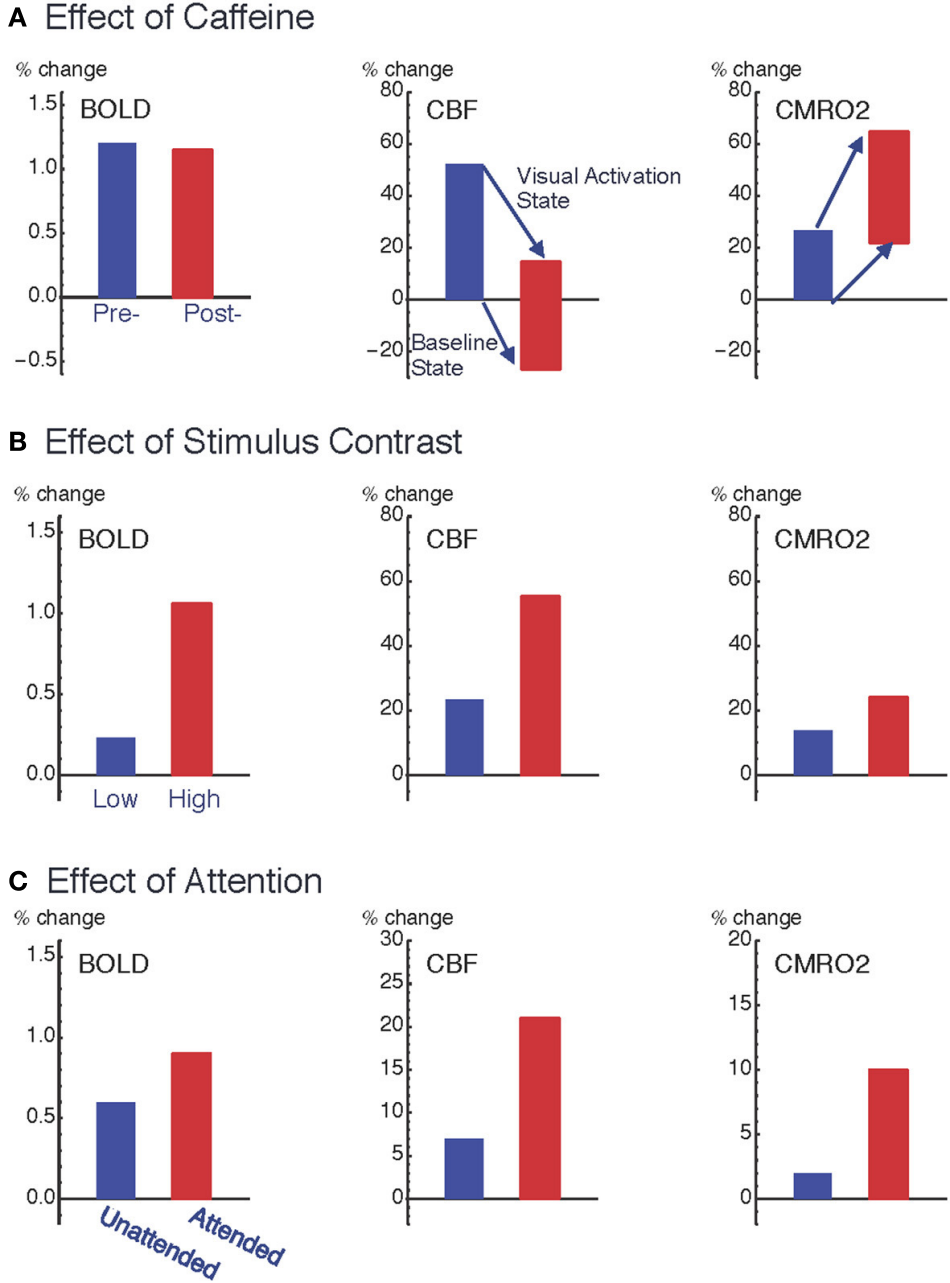
**Pattern of variation of the coupling ratio of CBF and CMRO_2_ responses**. Data from three studies of visual cortex show how responses are modulated by: **(A)** ingestion of 200 mg caffeine (Perthen et al., 2008; Griffeth et al., 2011); **(B)** increasing stimulus contrast (Liang et al., 2013); and **(C)** increasing attention to a fixed stimulus (Moradi et al., 2012). For the caffeine data **(A)**, changes are as a percentage of pre-caffeine baseline state, and the plots for CBF (middle column) and CMRO_2_ (right column) show both the baseline shift due to caffeine (the shift of the bottom of the bars) as well as the change in the activation state due to the visual stimulus response (the shift of the top of the bars). Note that the relative BOLD responses (left column) for the two conditions within each experiment (pre- vs. post-caffeine, low contrast vs. high contrast, and unattended vs. attended) do not quantitatively reflect the underlying CMRO_2_ response for those conditions. The BOLD response was unchanged with caffeine, despite a large change in the CMRO_2_ response to the stimulus, and the BOLD response greatly overestimated the CMRO_2_ change when stimulus contrast was changed and greatly underestimated the CMRO_2_ change when attention was modulated.

Caffeine provides a useful test for exploring the complexities involved in the BOLD response because it has both neural and vascular effects through inhibition of adenosine receptors, and thus affects multiple factors in Equation (1). Adenosine has the somewhat counterintuitive effect of inhibiting neural activity but increasing CBF, which is most likely a protective mechanism limiting O_2_ demand while trying to increase O_2_ delivery. We thus expect administration of caffeine to reduce CBF but potentially to increase CMRO_2_ as the effects of adenosine are blocked. In our study (Perthen et al., 2008; Griffeth et al., 2011) we used a calibrated BOLD experimental design that made it possible to refer all changes to the pre-caffeine baseline state, allowing us to look at both baseline changes due to caffeine and also the response to a visual stimulus before and after caffeine (Figure 2A). The primary findings were that baseline CBF was reduced by 25% due to caffeine, consistent with earlier studies (Chen and Parrish, 2009a), while baseline CMRO_2_ increased, and in addition the absolute CMRO_2_ response to the visual stimulus was increased by 60% post-caffeine [consistent with findings in (Chen and Parrish, 2009b)]. The latter result is consistent with the idea that caffeine led to increased excitability, in the sense that the same stimulus elicited a much stronger evoked response.

The surprising result, given these large changes in the underlying physiology, was that the BOLD response to the visual stimulus was unchanged by caffeine. The origin of this negative finding illustrates the complexity involved in interpreting the BOLD response, in this case because two effects were present but acting in opposite directions. The baseline shift, decreasing CBF with increasing CMRO_2_, would increase baseline levels of deoxyhemoglobin, creating a larger value of *A*. However, the increased neural excitability, with a larger change in CMRO_2_ compared to CBF in response to the visual stimulus, decreased the value of *n*. In our study population these two effects mutually cancelled, leaving the BOLD signal unchanged. In short, this example shows that large physiological changes, detected with quantitative fMRI methods, can be missed when looking only at BOLD responses.

## The variability of flow/metabolism coupling

The caffeine example raises a basic question: how variable is the CBF/CMRO_2_ coupling ratio under different conditions? For the past several years we have tried to address this question with a series of calibrated BOLD studies in human visual cortex. While we (Ances et al., 2008) and others (Chiarelli et al., 2007) have found different coupling ratios in different brain regions, our goal in these studies was to specifically test whether the coupling ratio changes for the same brain region under different conditions. For several conditions we found the coupling ratio *n* to be unchanged, in good agreement with earlier pioneering studies using the calibrated BOLD approach by Hoge et al. (1999). In particular, one scenario in which we expected to see coupling differences was comparing color and luminance stimuli designed to preferentially stimulate blob and interblob regions. Anatomically, these regions are defined by different concentrations of cytochrome oxidase, suggesting different capacities for oxidative metabolism. However, we found no evidence for a coupling difference when the stimuli were adjusted to evoke similar magnitudes of response (Leontiev et al., 2013).

However, in several other studies we found evidence for significant variability of the CBF/CMRO_2_ coupling ratio (Figure 2). In these studies we found that *n* was smaller for a weak stimulus compared with a stronger stimulus (varying contrast of the stimulus) (Liang et al., 2013), for an attended stimulus compared to the same stimulus when unattended (Moradi et al., 2012), and with adaptation to a sustained stimulus compared to the initial response (Moradi and Buxton, 2013). Put another way, compared to the CBF response these data are consistent with the CMRO_2_ response rounding off more as the stimulus intensity increases, responding more strongly to attention, and adapting more quickly to a sustained stimulus. Based on these studies an interesting empirical pattern is beginning to emerge for how CBF and CMRO_2_ respond to different types of neural activity. If the stimulus is modulated to create a stronger response (e.g., increasing stimulus contrast), CBF is modulated more than CMRO_2_ (*n* increases); on the other hand, if the brain state is altered such that the response to the same stimulus is increased (e.g., modulating attention, adaptation, or excitability with caffeine), CMRO_2_ is modulated more than CBF (*n* decreases). Because CBF and CMRO_2_ changes conflict in producing BOLD signal changes, this finding has an important implication for conventional BOLD-fMRI studies: the BOLD response exaggerates the effects of stimulus variation but is only weakly sensitive to modulations of the brain state that alter the response to a standard stimulus.

These effects are not small, as illustrated in Figure 2. Changing the stimulus contrast created a modest change in the evoked CMRO_2_ response but the BOLD response modulation was about twice as large. In contrast, attention created a large amplification of the CMRO_2_ response, with only a modest change in the BOLD response. Going back to our caffeine study, despite a large change in the CMRO_2_ response to the stimulus, there was no change in the BOLD response. In short, the BOLD signal could exaggerate the underlying change in CMRO_2_ or miss it entirely. Note that these effects are all consistent with our understanding of the conflicting effects of CBF and CMRO_2_ changes on the BOLD response, with relatively small changes in *n* having a large effect. The intriguing physiological phenomenon is that the coupling ratio is not fixed within a brain region, but varies under different conditions. This clearly presents a problem for the interpretation of the BOLD response alone, but these results also show that quantitative fMRI methods can provide a deeper probe of the physiology of brain activation, and raises the question: does the CBF/CMRO_2_ coupling ratio tell us something about the underlying evoked neural activity?

## Neural activity: what costs energy and what drives blood flow?

Our basic assumption is that CMRO_2_ is the physical parameter closest to the underlying neural activity in that it reflects the net energy cost of that activity. This assumption is important to make explicit, because it is complicated by the dissociation of glucose metabolism and oxygen metabolism in the brain (Fox et al., 1988). For reasons that are not well understood, glucose metabolism increases more than oxygen metabolism with increased neural activity. Nevertheless, most of the energy required in terms of adenosine triphosphate (ATP) generation to support the neural activity is thought to come from oxidative metabolism of pyruvate, with the contribution from glycolysis as a small fraction (Buxton and Frank, 1997; Lin et al., 2010).

The primary energy cost of neural activity is the restoration of sodium and calcium gradients partially degraded by neural activity (Attwell and Laughlin, 2001; Buxton, 2013). These ions are maintained in a state far from thermodynamic equilibrium, with high extracellular concentrations and low intracellular concentrations. An action potential arriving at an excitatory synapse triggers a chain of events that leads to the opening of sodium channels on the post-synaptic dendrite. The sodium then flows through the channel due to the electrochemical gradient, creating an excitatory inward synaptic current that partially depolarizes the membrane potential. This in turn leads to the opening of voltage sensitive calcium channels, creating an influx of calcium ions (Lauritzen, 2005). If the net excitatory current into the post-synaptic cell reaches the soma with sufficient strength an action potential is generated. Importantly, none of this signaling process requires energy, because each step is downhill in a thermodynamic sense. The energy cost is in restoring the ion gradients by pumping sodium and calcium back out of the cell, requiring ATP as the source of free energy for this thermodynamically uphill process. For this reason, excitatory neural activity has a high energetic cost. While there is an energy cost associated with clearing neurotransmitter from the synaptic cleft and repackaging it in the pre-synaptic terminal, this is thought to be less than 10% of the total energy cost of synaptic activity (Attwell and Laughlin, 2001). There is also a cost in generating and propagating the action potential, and although this cost is estimated to be about half of the energy cost for the rat brain, the higher number of synapses each axon projects to in the primate brain shifts the dominant energy cost to recovery from synaptic activity rather than action potential production. Estimates for the primate brain are that excitatory synaptic activity accounts for about 3/4 of the energy costs of neural signaling (Attwell and Iadecola, 2002).

Inhibitory synaptic activity is likely to have a much lower energy cost. Inhibitory activity can take several forms, but the simplest is the opening of chloride channels. The extracellular medium has a higher concentration of both sodium and chloride than the intracellular medium. However, because chloride ions are negatively charged, their distribution is close to equilibrium with the negative intracellular electric potential. The membrane potential reflects the balance of open channels for different ions, and opening more chloride channels tends to peg the membrane potential at the chloride equilibrium potential, effectively reducing the effect of simultaneous excitatory sodium currents. When GABA, the primary inhibitory neurotransmitter in the cortex, is released there will again be the energy cost associated with clearing and repackaging the neurotransmitter, but there is no large energy cost for post-synaptic ion pumping: chloride ions are already in a near equilibrium distribution, and there is no large sodium flux as there is for excitatory activity.

Blood flow is driven strongly by aspects of excitatory synaptic activity, a well-matched feed-forward system given that the dominant energy cost is excitatory activity. In contrast, the role of inhibitory interneurons in the control of CBF presents an intriguingly complex picture (Cauli et al., 2004). Some classes of interneurons have a constricting effect on blood vessels, acting to reduce CBF. However, other classes of interneurons have a vasodilatory effect, increasing CBF. In particular, one of the most potent vasodilators known, nitric oxide (NO), is released by inhibitory interneurons (Estrada and DeFelipe, 1998). As with the effects of adenosine, discussed above in the context of our caffeine experiment, this is an example of an agent that has opposite effects on CBF and CMRO_2_: acting to increase CBF while also acting to inhibit neural activity and thus reduce CMRO_2_.

## Does CBF/CMRO_2_ coupling reflect the balance of inhibitory and excitatory neural activity?

The observation that there are examples of inhibitory mechanisms that have a larger effect on increasing CBF than on increasing CMRO_2_ (or even act to reduce CMRO_2_) suggests a speculative hypothesis: the coupling ratio *n* of CBF and CMRO_2_ responses to a stimulus tracks with the ratio of inhibitory to excitatory activity in the neural response. In this picture, when there is a strong involvement of inhibitory activity, CBF is increased relative to CMRO_2_ because of the vasodilatory effect of the inhibitory mechanisms, and thus *n* is larger. In our experiments we had no direct information on the balance of excitatory and inhibitory activity, but we can imagine plausible scenarios based on this hypothesis. For our attention experiment, the visual stimulus was either the focus of the task or a distractor for another task the subject was asked to perform; we hypothesize that inhibition of the response to the stimulus in the latter unattended case would lead to a larger *n*, as observed (Moradi et al., 2012). With adaptation, we hypothesize that increased involvement of inhibitory mechanisms over time would act to reduce the CMRO_2_ response while continuing to push up the CBF response, as observed (Moradi and Buxton, 2013). In the caffeine experiment, before caffeine was given adenosine was more effective, tending to increase the balance of inhibitory and excitatory activity and boost the CBF response but suppress the CMRO_2_ response (Griffeth et al., 2011). With increasing contrast of a visual stimulus, animal studies of the behavior of different cellular types found a flattening of the response of simple regularly spiking neurons (thought to be glutamatergic excitatory cells) but continued increasing activity of simple fast spiking neurons (thought to be inhibitory GABAergic neurons) (Contreras and Palmer, 2003), suggesting a greater proportional involvement of inhibitory activity as contrast increases, consistent with our finding of increased *n* (Liang et al., 2013).

This hypothesis is speculative, but suggests the possibility of a new direction in which quantitative fMRI may be able to provide information on the underlying activity. Note that this information is in addition to the magnitude of the overall evoked response, as reflected in the CMRO_2_ response. The overall response depends on the balance of excitatory and inhibitory activity in a nonlinear way, and the overall response magnitude (the CMRO_2_ response) could be large for either a weaker stimulus with no inhibition or a stronger stimulus with more involvement of inhibitory mechanisms. If this hypothesis is true, then the ratio of CBF and CMRO_2_ responses could provide an index of the involvement of inhibitory neural activity that could distinguish these cases.

In conclusion, the BOLD response is a complex phenomenon, and the magnitude of the BOLD response cannot be taken as a quantitative reflection of underlying activity. Our studies suggest a pattern in which the BOLD magnitude exaggerates the physiological changes when the stimulus strength is changed, but underestimates or completely misses those changes when the brain state is modulated to change the response to the same stimulus. This is a problem for interpreting BOLD imaging alone, but quantitative fMRI methods offer a way to untangle the ambiguities of the BOLD response. Current work in our group is focused on developing approaches to apply these methods to analyze dynamic responses (Simon et al., 2013) and to make the calibration easier to apply by eliminating the need to breathe special gas mixtures (Blockley et al., 2012). Potentially, quantitative fMRI methods provide two candidate measurements of neural activity: the overall evoked response, as reflected in the CMRO_2_ change; and the balance of evoked inhibitory and excitatory activity, as reflected in the coupling ratio of the CBF and CMRO_2_ responses. We emphasize though, that this picture is speculative, based on two elements: (1) a limited set of experiments in human primary visual cortex to explore the variability of CBF/CMRO_2_ coupling; (2) limited understanding of the role of inhibitory mechanisms on CBF control (most of which comes from brain slice experiments, rather than *in vivo* experiments) and very little understanding of effects of inhibitory activity on CMRO_2_ (although the theoretical arguments are plausible). Each of these elements requires much more experimental attention to test whether there is any truth in this speculative hypothesis.

### Conflict of interest statement

The authors declare that the research was conducted in the absence of any commercial or financial relationships that could be construed as a potential conflict of interest.
