# Corrigendum: Variability of the coupling of blood flow and oxygen metabolism responses in the brain: a problem for interpreting BOLD studies but potentially a new window on the underlying neural activity

**DOI:** 10.3389/fnins.2014.00241

**Published:** 2014-09-23

**Authors:** Richard B. Buxton, Valerie E. M. Griffeth, Aaron B. Simon, Farshad Moradi, Amir Shmuel

**Affiliations:** ^1^Department of Radiology, Center for Functional MRI, University of CaliforniaSan Diego, La Jolla, CA, USA; ^2^Departments of Neurology and Neurosurgery, Physiology and Biomedical Engineering, Montreal Neurological Institute Brain Imaging Centre, McGill UniversityMontreal QC, Canada

**Keywords:** fMRI, BOLD, cerebral blood flow, cerebral metabolic rate of oxygen, inhibition

Through an oversight the author list of the published version of this paper failed to reflect the important contributions of Amir Shmuel. For all aspects of scientific attribution he should be considered to be the final author on the paper, so that the appropriate author list is: Richard B. Buxton, Valerie E. M. Griffeth, Aaron B. Simon, Farshad Moradi, and Amir Shmuel.

## Author contributions

Richard B. Buxton and Amir Shmuel conceptualized the hypothesis that CBF and CMRO_2_ may be driven differentially by excitatory and inhibitory activity and formulated experiments to test the idea. Valerie E. M. Griffeth, Aaron B. Simon, and Farshad Moradi conducted experiments and contributed ideas on how their results could be interpreted within the hypothesis. Richard B. Buxton wrote the manuscript, and all authors contributed to editing the final version.

### Conflict of interest statement

The authors declare that the research was conducted in the absence of any commercial or financial relationships that could be construed as a potential conflict of interest.

